# Fault Feature Extraction of Hydraulic Pumps Based on Symplectic Geometry Mode Decomposition and Power Spectral Entropy

**DOI:** 10.3390/e21050476

**Published:** 2019-05-07

**Authors:** Zhi Zheng, Ge Xin

**Affiliations:** 1 College of Mechanical Engineering, North China University of Science and Technology, Tangshan 063210, China; 2 School of Traffic and Transportation, Beijing Jiaotong University, Beijing 100044, China; 3 Beijing Research Center of Urban Traffic Information Sensing and Service Technologies, Beijing Jiaotong University, Beijing 100044, China

**Keywords:** hydraulic pump, symplectic geometry mode decomposition, power spectral entropy

## Abstract

Aiming at fault feature extraction of a hydraulic pump signal, a new method based on symplectic geometry mode decomposition (SGMD) and power spectral entropy (PSE) is proposed. First, the SGMD is applied to decompose a multi-component fault signal, then the *N* symplectic geometry components (SGCs) can be obtained. Second, the *N* SGCs are reconstructed as a signal of interest and, consequently, the power spectral entropy of each constructed signal is computed to quantify the complexity and uncertainty of their spectra. Finally, the difference value (D-value) between the adjacent entropies is used as a SGCs criterion, whose turning point indicates the most information of reconstructed signal. Hydraulic pump signals are tested and verified, and results demonstrate that the proposed method can extract the richest fault feature information of hydraulic pump signals effectively.

## 1. Introduction

Hydraulic pumps are very important parts in a hydraulic system. As a power source, it converts mechanical energy into pressure energy. In the real world, it is widely used in mechanical equipment, e.g., space shuttles, airliners, rolling mills, refining machinery and cranes, etc. Due to the extreme running environment, i.e., high temperature, high pressure and heavy load, the health conditions of a pump degrade over time. In practice, its fault feature information is often contaminated by interferences and noises, thus the diagnosis of hydraulic pumps has attracted constant interest in the scientific community [1,2,3,4,5].

The wavelet transform (WT), which localizes the signature in the time and frequency domain, is widely used in fault feature extraction of vibration signals [6,7,8,9]. A signal can be decomposed into several various frequency bandwidth or scale signals with different resolutions. The WT is also the Fourier transform (FT) of adjustable window, thus it has the shortcomings of the FT. Since the scale factor and wavelet basis function is selected before decomposition, it is not adaptive to match the morphological feature information of signals. Meanwhile the window length of wavelet basis function is fixed, thus energy leakage is produced [10,11].

Principal component analysis (PCA) is simple and efficient in processing a linear signal with high correlations, which has been used in fault diagnosis. A main limitation of PCA is that the PCA model is time-invariant, while some real processes are time-varying and with normal process changes. As a result, higher-order nonlinear feature information may be lost in the case of process monitoring with normal changes and drifts [12,13].

Empirical mode decomposition (EMD) is an effective method to process a nonlinear signal [14,15]. Based on the morphological feature information of a multi-component signal, the signal can be decomposed into several single-component signals by EMD, thus the EMD is an adaptive tool and independent of user-defined parameters and the preset function. Decomposition processing is based on applying cubic splines to fit lower and upper envelope of morphological feature information, which greatly leads to mode mixing and overdecomposition [14,15,16,17,18,19].

Based on EMD, Smith proposed local mean decomposition (LMD), and it is an effective and adaptive time-frequency analysis tool [20]. Different from EMD, LMD can decompose a multi-component signal into a series of mono-components, and they are called product functions (PFs), and each PF is an amplitude-modulated and frequency-modulated signal (AM-FM signal). It is noteworthy that LMD performs better than EMD at mode mixing, end effect, computation cost and so on [21].

Singular spectrum analysis (SSA) is a method based on singular value decomposition [22,23]. The SSA mainly consists of embedded dimension, singular value decomposition and reconstruction of components, thus construction subjectivity of trajectory matrix has a great influence on the processing result. As a result, it is not a good tool to study a nonlinear and noisy signal [22,23,24,25].

To address the shortcomings of the above methods, symplectic geometry mode decomposition (SGMD) is proposed based on the sympletic geometry algorithm [26,27]. It was first introduced into fault diagnosis in 2018 [28]. A symplectic geometry similarity transformation is applied to the signal, and the Hamiltonian matrix can be obtained. Eigenvalues and eigenvectors of the matrix can be computed, then several single-component signals can be constructed by the eigenvectors, and the single-component signal is called symplectic geometry components (SGCs). SGMD is good at preserving the structure features of the system. However, the number of SGCs is proportional to the signal length, which probably leads to high computational cost, even overdecomposition.

In order to measure information amount quantitatively, information entropy is proposed based on probability theory and mathematical statistics, and it has been introduced into information theory [29]. Thus, power spectral entropy (PSE) is proposed to indicate the spectral distribution and quantitatively measure energy distribution of a signal in the frequency domain, which explains the spectrum composition of vibration signal. The more uniformity the vibration energy distributes in the whole frequency composition, the less complex the signal is [30,31,32].

In this study, a new scheme to extract the fault feature of a hydraulic pump signal is proposed as follows. The signal is first decomposed by SGMD, then the *N* SGCs can be obtained to reconstruct the signal of interest. Since the PSE can quantitatively measure information amount, the number of SGCs can be selected by the difference value (D-value) between the adjacent entropies, while suppressing the interferences and noises.

The rest of the paper is organized as follows: in Section 2, the algorithm of SGMD is introduced. In Section 3, the PSE is presented. In Section 4, it shows the flow chart of the proposed method. In Section 5, it demonstrates the experimental decomposition results of the hydraulic pump fault signals by using the proposed method. In Section 6, the conclusions of this investigation are summarized.

## 2. Algorithm of SGMD

Algorithm of SGMD is described as follows [26,27,28]:

(1) Phase space reconstruction

Let *s* = (*x*_1_, *x*_2_, …, *x_n_*) be a one-dimensional discrete signal, and reconstruction matrix *X* can be written as:

(1) $$X=\left[\begin{array}{ccccc} x_{1} & x_{1+\tau } & . & . & x_{1+(d-1)\tau }\\ x_{2} & x_{2+\tau } & . & . & x_{2+(d-1)\tau }\\ . & . & . & . & .\\ . & . & . & . & .\\ x_{m} & x_{m+\tau } & . & . & x_{m+(d-1)\tau } \end{array}\right]$$

Let *m* = *n* − (*d* − 1)*τ*, where *d* is the embedding dimension, *τ* is the delay time. Appropriate values of *d* and *τ* are selected to get the corresponding *X*. However, the value of *d* has a great influence on *X*. If the normalized frequency of *s* is less than the given threshold 10^−3^, thus *d* is set to *n*/3, where *n* is the length of s. Otherwise, the *d* is set to 1.2 × (*F*_s_/*f*_max_), where *F*_s_ is sampling frequency [28].

(2) QR decomposition of symplectic orthogonal matrix 

For constructing Hamiltonian matrix *M*, autocorrelation analysis of the trajectory matrix is applied to get covariance symmetric matrix *A* = *X*^T^*X*, thus *M* can be defined as:

(2) $$M=\left[\begin{array}{cc} A & 0\\ 0 & -A^{T} \end{array}\right]$$

Let *N* = *M*^2^, thus *M* and *N* are both Hamilton matrices. Symplectic orthogonal matrix is constructed as *Q*, namely:

(3) $$Q^{T}NQ=\left[\begin{array}{cc} B & R\\ 0 & B^{T} \end{array}\right]$$

where *B* is upper triangular matrix, *b_ij_* = 0 (*i* > *j* + 1). B can be obtained by applying Schmidt orthogonalization to matrix *N*, and their eigenvalues are *λ*_1_, *λ*_2_, …, *λ_d_* respectively. Let $\sigma_{i}=\sqrt{\lambda_{i}}$. *Q_i_* corresponds to *σ_i_* and is the eigenvalues of matrix *A*. Thus matrix *X_i_* = *Y*^T^ can be obtained based on $S_{i}=Q_{i}^{T}X_{}^{T}$ and $Y=Q_{i}^{}S_{i}$, *X* is consisted of *d* as single-component and expressed as:

X = X_1_ + X_2_ + ... + X_d_ (4)

(3) Diagonal averaging transformation

*X_k_* (1 ≤ *k* ≤ *d*) can be transformed into a time series with length *n*, and sum of *d* time series is original time series *s*.

For any initial single component matrix *X_i_*, let *X_m_*_×*d*_ = (*x_ij_*)_*m*×*d*_, where *x_ij_* is the elements of the matrix, 1 ≤ *i* ≤ *d*, 1 ≤ *j* ≤ *m*, d* = min(*m*, *d*), m* = max(*m*, *d*) and *n* = *m* + (*d* − 1)*τ*. Let x_ij_* = x_ji_* if *m* < *d*, otherwise x_ij_* = *x_ij_*. Thus, *y_k_* can be obtained:

(5) $$y_{k}=\{\begin{array}{cc} \frac{1}{k}\sum_{p=1}^{k}y_{p,k-p+1}^{*} & \;1\leq k\leq d^{*}\\ \frac{1}{d}\sum_{p=1}^{d^{*}}y_{p,k-p+1}^{*} & \;d^{*}\leq k\leq m^{*}\\ \frac{1}{n-k+1}n\sum_{p=k-m^{*}+1}^{n-m^{*}+1}y_{p,k-p+1}^{*} & \;m^{*}<k\leq n \end{array}$$

Based on Equation (5), *X_k_* can be transferred into a time series *Y_i_* (*y*_1_, *y*_2_, …, *y_n_*) by diagonal averaging, thus s can be decomposed into *d* single-component called SGCs, and they are expressed as:

*Y* = *Y*_1_ + *Y*_2_ + … + *Y*_d_ (6)

## 3. Principle of PSE

PSE is able to indicate spectral distribution and quantify energy distribution of a signal in the frequency domain. If spectrum distribute uniformly, PSE value is big, which means that complexity and uncertainty is high.

Information entropy can be defined as follows [30,31,32]: for a one-dimensional discrete signal *s* = (*x*_1_, *x*_2_, …, *x_n_*), its corresponding probability can be defined as:

*P* = {*p*_1_, *p*_2_, …, *p_n_*}   0 ≤ *p_i_* ≤ 1, *i* = 1, 2, …, *n* (7)

where *p_i_* is under constraints of $\sum_{i=1}^{n}p_{i}=1$.

Thus, the information entropy can be written as:

(8) $$E=-\overset{n}{\underset{i=1}{\sum }}P_{i}\ln P_{i}$$

Based on information entropy, FFT transform is applied to *s*, and PSE can be computed based on the spectral distribution in the frequency domain, thus the algorithm of PSE is summarized as follows:

For the signal *s*, its power spectrum estimation can be defined as:

(9) $$\hat{S}\left(\omega \right)=-\frac{1}{n}{\left|\overset{n}{\underset{i=1}{\sum }}x_{i}e^{-j\omega i}\right|}^{2}$$

Next, it can be redefined as:

(10) $$\hat{S}\left(\omega \right)=-\frac{1}{n}{\left|X\left(\omega \right)\right|}^{2}$$

where *X*(*ω_i_*) is FFT transform result of *s*, *ω_i_* is one of spectra.

According to Parseval’s theorem, energy is conserved in transforming from the time domain to the frequency domain, thus it can be written as:

(11) $$\overset{n}{\underset{i=1}{\sum }}{\left|x_{n}\right|}^{2}=\overset{n}{\underset{r=1}{\sum }}{\left|S\left(r\right)\right|}^{2}$$

*S_r_* (*r* = 1, 2, …, *n*) can be seen as energy partition of *s* in the frequency domain, so PSE can be defined as:

(12) $$PSE=-\overset{n}{\underset{r=1}{\sum }}P_{r}\ln P_{r}$$

where *p_r_* is the proportion of *r*-th spectrum energy in all spectrum energy, and it also means the proportion probability, which can be written as:

(13) $$p_{r}=S_{r}/\sum_{r=1}^{n}S_{r}$$

## 4. Flow Chart of the Method Based on SGMD and PSE

**Figure 1:**
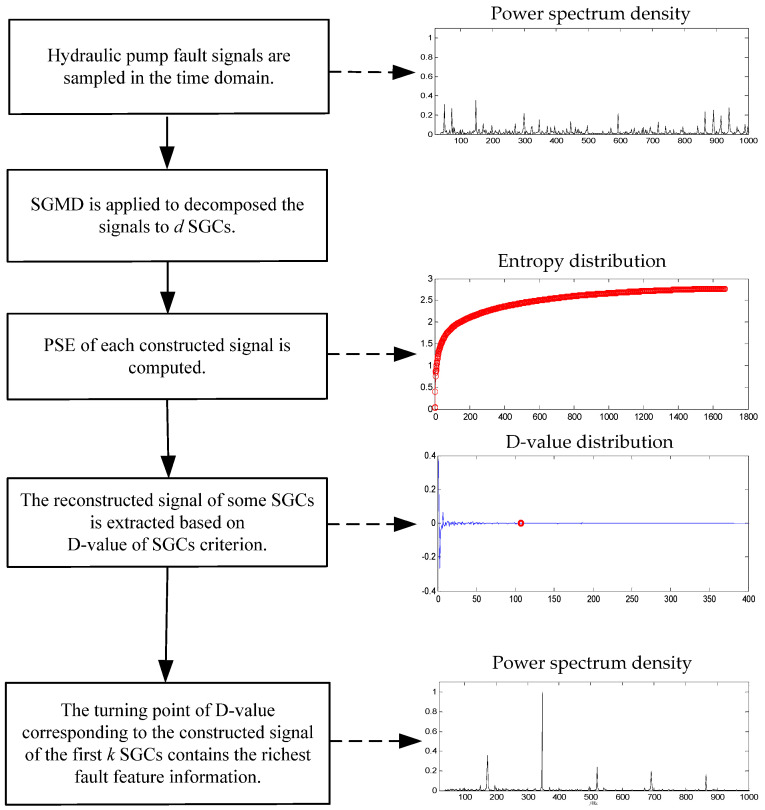
Flow chart of the method.

A hydraulic pump fault signal is sampled with the length of *n* points. SGMD is applied to decompose the signal, and ⌊*n*/3⌋ = *d* SGCs can be obtained, then the first *k* (*k* = 1, 2, …, *d*) SGCs are constructed, respectively, and *d* constructed signals can be obtained. The PSE of each constructed signal is computed, and PSE value of the former constructed signal subtracted from that of the latter one is defined as a difference value (D-value), and the value is chosen as turning point if D-values is very close to 0, and the turning point implies that the constructed signal of the first *k* SGCs contains the richest fault feature information. The flow chart of the proposed method is shown in Figure 1.

## 5. Application to Hydraulic Pump Fault Signals

### 5.1. Experimental Scheme

For the sake of verifying the effectiveness of the proposed method, an experiment is implemented on a swashplate axial plunger pump. The rotational speed and outlet pressure of the pump are 1470 r/min and 10 MPa, respectively. Rotational frequency is 1470/60 = 24.5 Hz, and the pump has seven slippers. If one of swashplates wears, loose slipper and center spring wear faults happen, the hydrostatic bearing balance between the slipper and swashplate will be broken, thus the force of the slipper impacting on the swashplate will become large. In addition, flow pulsations on the swashplate caused by the plunger (the slipper and the plunger are in one part) will increase, thus both the force and pulsations will cause considerable vibration on the swashplate. One sensor is placed on the cover (the cover and the swashplate are in one part), the vibration fault signal is sampled by a sensor and contains a large amount of feature information. The fault feature frequency of swashplate wear is a rotational frequency of 24.5 Hz. Since the pump has seven slippers, the fault feature frequency of the loose slipper and center spring wear are both 24.5 × 7 = 171.5 Hz [33].

**Figure 2:**
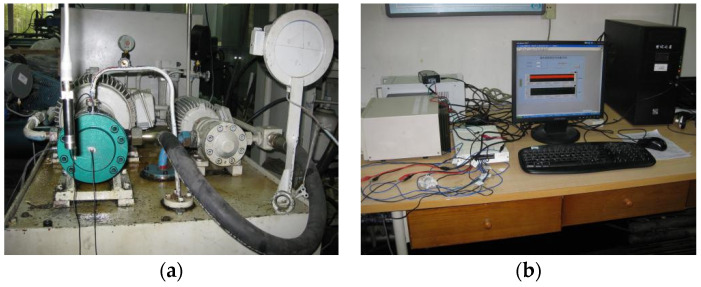
Test-rig of swashplate axial plunger pump. (**a**) Experimental system swashplate axial plunger pump; (**b**) Data acquisition equipment.

The experimental system is shown in Figure 2.

### 5.2. Normal Signal

**Figure 3:**
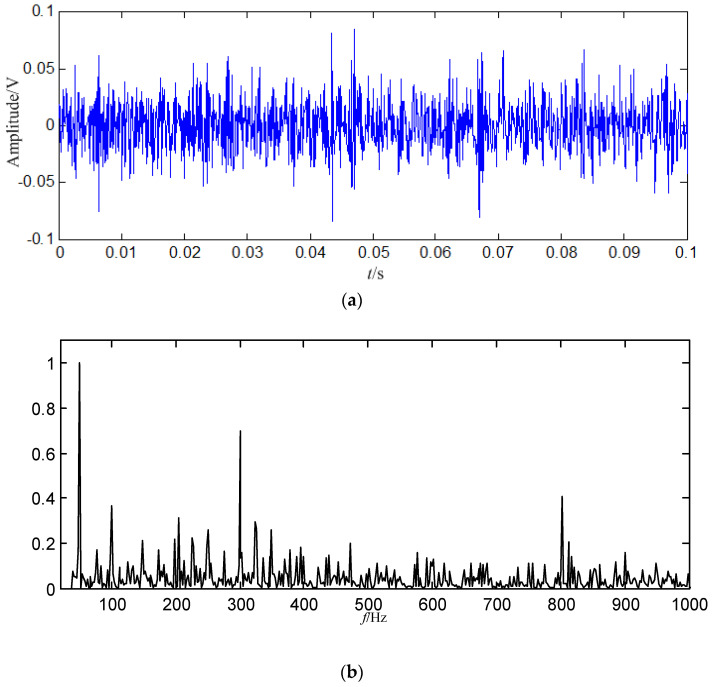
Normal signal. (**a**) Waveform in the time domain; (**b**) Power spectrum density in the frequency domain.

In order to demonstrate the fault feature extraction ability of the proposed method, the normal signal of 0.1 s is shown in Figure 3.

### 5.3. Application to Swashplate Wear Fault Signal

#### 5.3.1. Application to Swashplate Wear Fault Signals Based on SGMD

**Figure 4:**
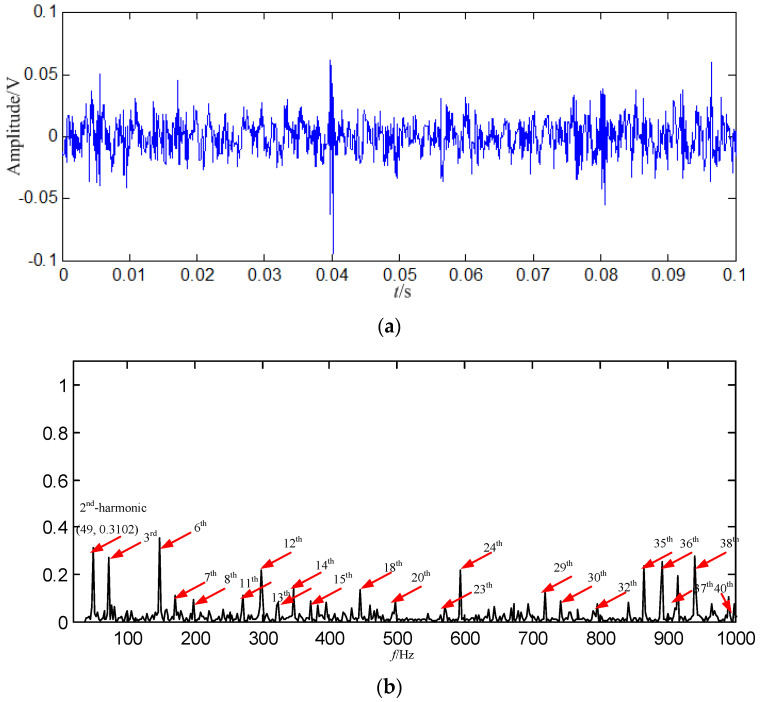
Swashplate wear fault signal. (**a**) Waveform in the time domain; (**b**) Power spectrum density in the frequency domain.

A swashplate wear fault signal with 5000 points (0.5 s) is decomposed in this study. In order display the signal clearly, the signal of 0.1 s is shown in Figure 4.

**Figure 5:**
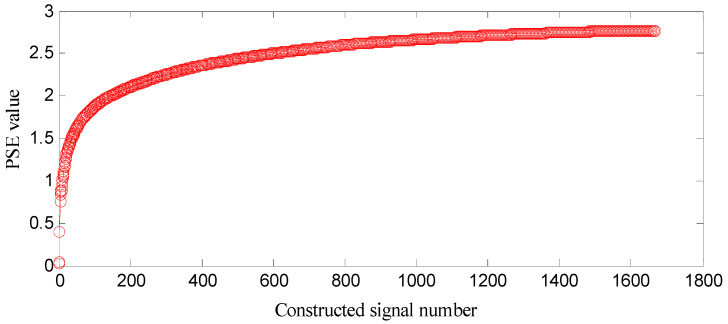
Entropy distribution based on swashplate wear fault signals.

SGMD is applied to the decomposed swashplate wear fault signal, and SGCs of ⌊*d*⌋ = *n*/3 = 1666 can be obtained. The first *k* SGCs are constructed, respectively, and 1666 constructed signals can be obtained, where *k* = 1, 2, …, 1666. In order to select some SGCs which contain the richest fault feature information, the SGCs criterion based on *PSE* is proposed. The *PSE* of each constructed signal is computed, and the entropy distribution is displayed in Figure 5.

In Figure 5, a few SGCs corresponding to the front range of small *PSE* value are constructed, which means that some of fault feature information can be extracted at the fault feature frequency and its harmonics. With more SGCs being constructed, all of fault feature information can be extracted and the amount of information becomes large, which implies that there will be a lot of spectra and the spectral value becomes big at these frequencies, thus spectrum distribution becomes more uniform, and complexity and uncertainty of the distribution is high, thus the *PSE* value becomes big. In the hind range of a big *PSE* value, more SGCs means that more noise information can be included, and there will be a lot of spectra and their values become big at other frequencies, thus spectral distribution becomes much more uniform, and result shows that *PSE* value increases and is nearly stable.

**Figure 6:**
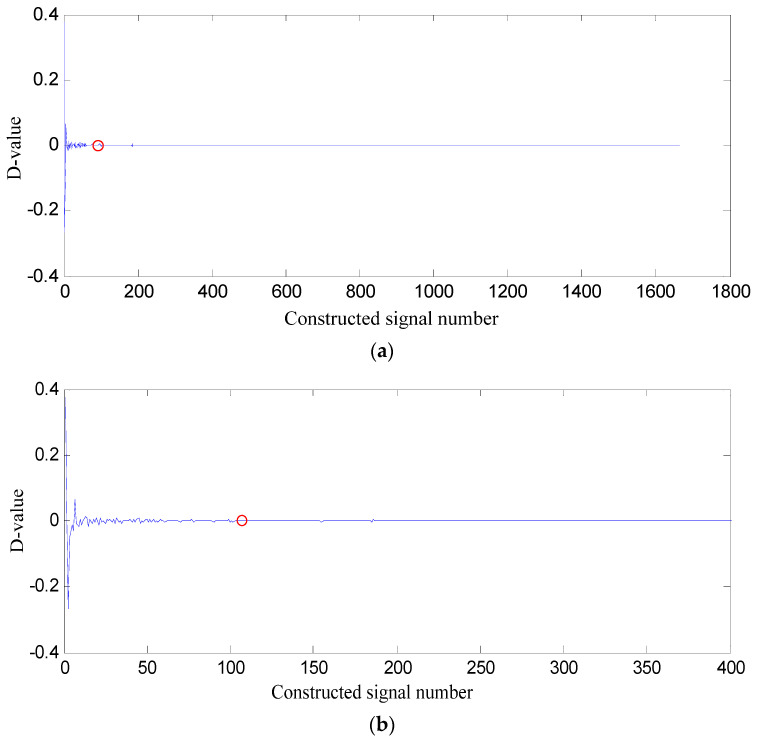
D-value distribution based on swashplate wear fault signal. (**a**) All of constructed signal number; (**b**) The first 400 constructed signals.

*PSE* value of the former constructed signal subtracted from that of the latter one is difference value (D-value), and the D-value distribution is displayed in Figure 6.

In Figure 6, D-values are very different in [0 128], and the values in [129 1665] are very close to 0, thus 128 is the turning point. The *PSE* value corresponding to 128 is not big and in the front range of *PSE* value, which means that the constructed signal of the first 128 SGCs may contain the richest fault feature information.

**Figure 7:**
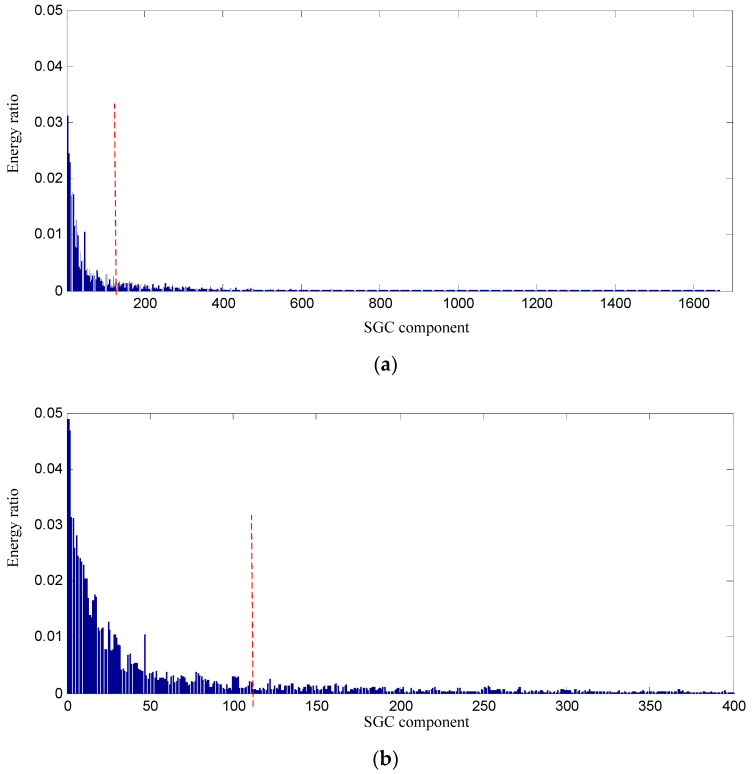
Energy distribution of each SGC component based on swashplate wear fault signal. (**a**) All of components; (**b**) The first 400 components.

In order to testify the effectiveness of the proposed SGCs criterion method, the energy ratio of SGC is adopted. The energy of each SGCs and the total energy of all components are computed, and the energy ratio of each component energy to total energy can be obtained, and their distribution of 1666 components is displayed in Figure 7.

**Figure 8:**
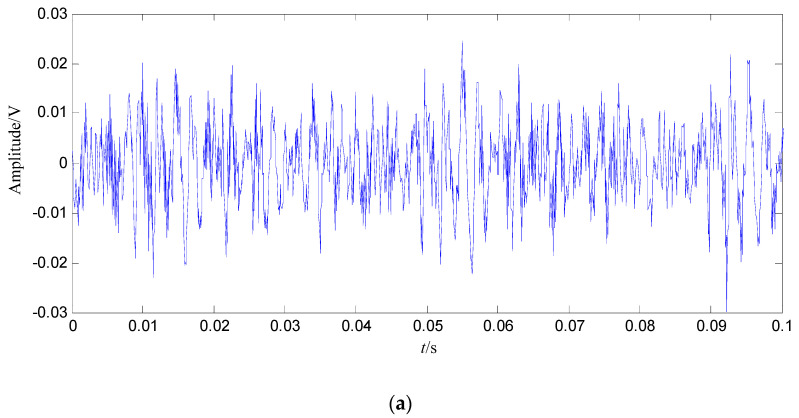

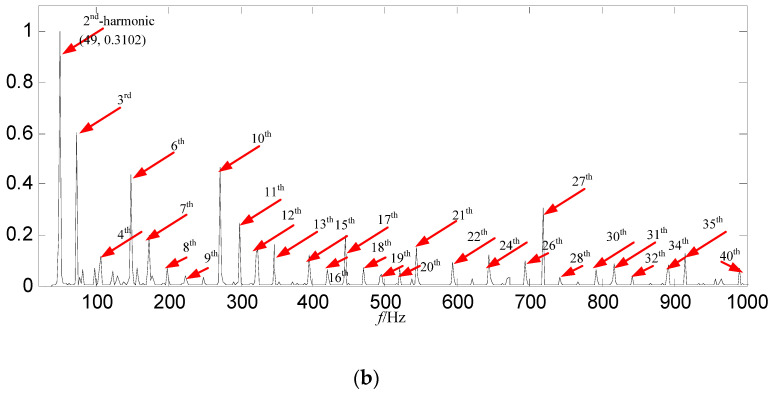
The constructed signal based on swashplate wear fault signal. (**a**) Waveform in the time domain; (**b**) Power spectrum density in the frequency domain.

Conclusions can be drawn from the above, namely that the constructed signal of the first 128 SGCs nearly involves all the energy, and thus the constructed signal contains the richest fault feature information. The waveform and power spectrum density of the constructed signal in the time and frequency domain are shown in Figure 8.

In Figure 8b, the fault feature information is at fault feature frequency of 24.5 Hz and its harmonics are all extracted, and their amount are very large, and there are nearly no interference components in other frequencies. Compared with the swashplate wear fault signal in Figure 4b and normal signal in Figure 3b, the amount of fault feature information extracted from the constructed signal (the first 128 SGCs) is much larger.

#### 5.3.2. Application to Swashplate Wear Fault Signal Based on EMD

**Figure 9:**
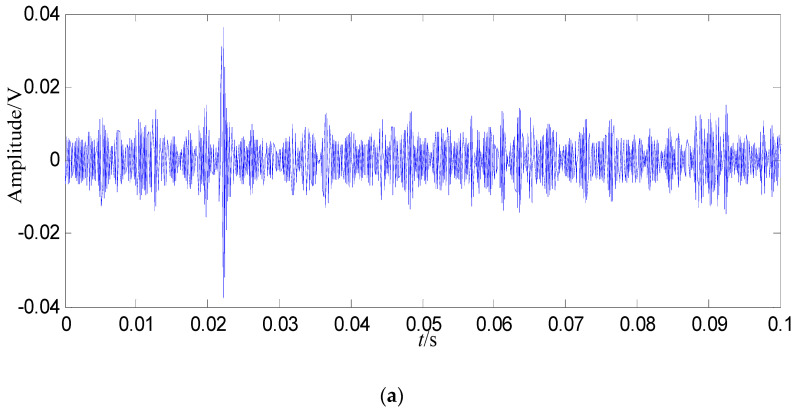

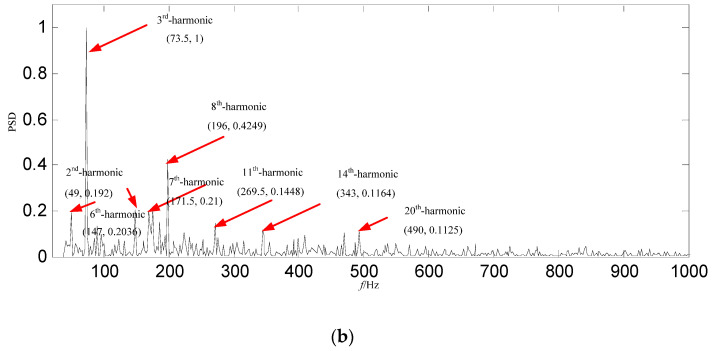
The EMD result based on swashplate wear fault signal. (**a**) Waveform of *IMF*_1_ in the time domain; (**b**) Power spectrum density of *IMF*_1_ in the frequency domain.

In order to demonstrate the effectiveness and advantages of the proposed method, the signal is also decomposed by EMD. Energy of the first intrinsic mode function (IMF_1_) is the biggest among all IMFs, thus IMF_1_ is selected as the data source, and IMF_1_ is shown in Figure 9.

In Figure 9b, most of the fault feature information is at a fault feature frequency of 24.5 Hz and its harmonics are not extracted, and the amount of most of the fault feature information is very small, and there are a lot of interference components at other frequencies. Compared with the constructed signal in Figure 8b, the fault feature information amount is less than those obtained by SGMD.

#### 5.3.3. Application to Swashplate Wear Fault Signal Based on LMD

**Figure 10:**
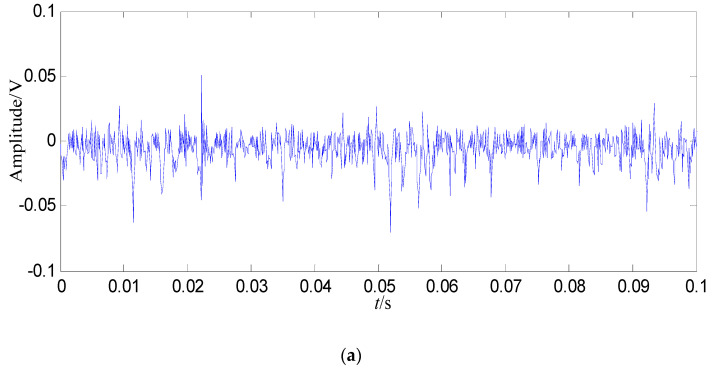

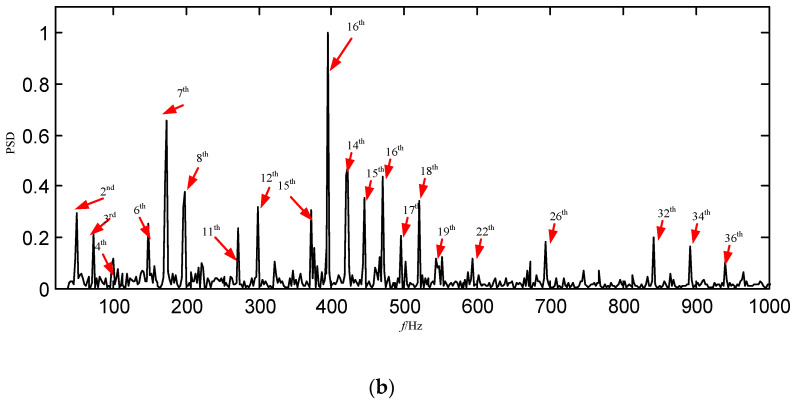
The LMD result based on swashplate wear fault signal. (**a**) Waveform of *PF*_1_ in the time domain; (**b**) Power spectrum density of *PF*_1_ in the frequency domain.

The signal is also decomposed by LMD, and some PFs can be obtained. Among above PFs, energy of PF_1_ is the biggest after analysis, therefore PF_1_ is used as data sources, and it is shown in Figure 10.

In Figure 10b, the fault feature information is extracted at a fault feature frequency of 24.5 Hz and some of its harmonics, and there are also many interference components at other frequencies. Compared with the SGMD result in Figure 8b and EMD result in Figure 9b, the fault feature information amount is less than those obtained by SGMD, and more than the EMD result.

Some conclusions can be drawn from the above: (1) the swashplate wear fault signal can be decomposed by SGMD effectively, and the first 128 SGCs which contain the richest fault feature information can be selected by the SGCs criterion. The fault feature information amount amount by SGMD is more than those obtained by EMD and LMD, thus the proposed method performs better than EMD and LMD. (2) Compared with the normal signal in Figure 3a, swashplate wear fault signal in Figure 4a, *IMF*_1_ in Figure 9a and *PF*_1_ in Figure 10a, although the fault signal, *IMF*_1_ and *PF*_1_ have periodic impact feature information in time domain waveform, they are caused by interferences and noises.

### 5.4. Application to Loose Slipper Fault Signal

#### 5.4.1. Application to Loose Slipper Fault Signal Based on SGMD

**Figure 11:**
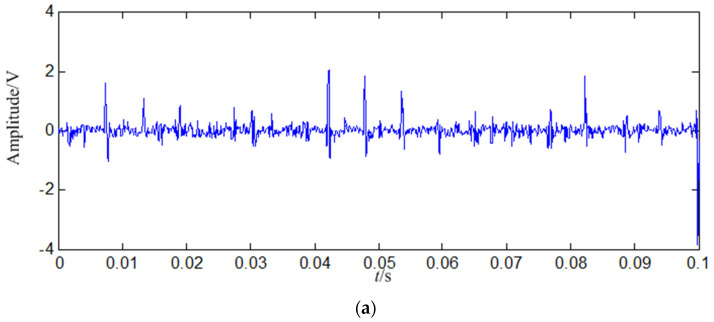

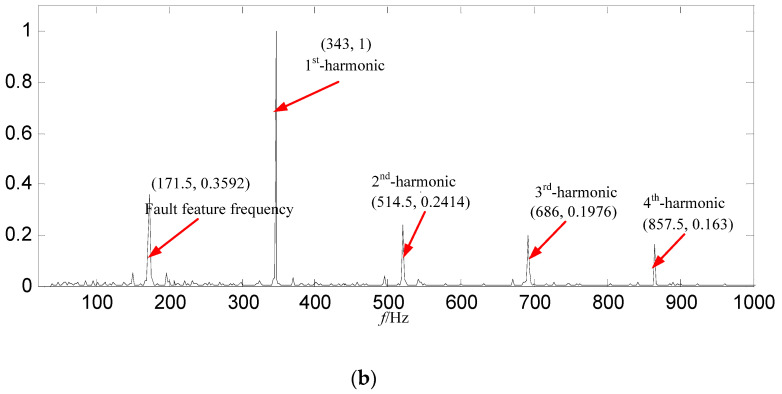
Loose slipper fault signal. (**a**) Waveform in the time domain; (**b**) Power spectrum density in the frequency domain.

A loose slipper fault signal with 5000 points (0.5 s) is decomposed in this study. In order to display the signal clearly, the signal of 0.1 s is shown in Figure 11.

**Figure 12:**
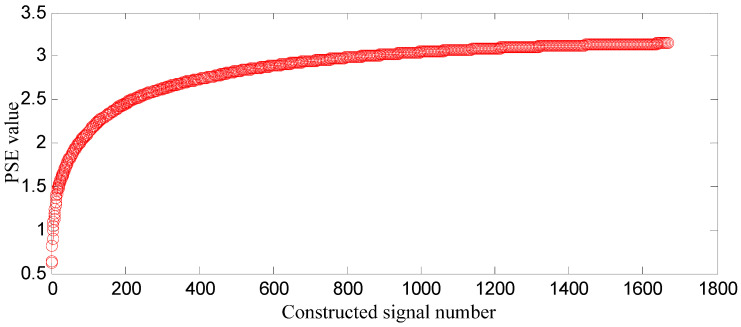
Entropy distribution based on loose slipper fault signal.

SGMD is applied to decompose the loose slipper fault signal, and SGCs of ⌊*d*⌋ = *n*/3 = 1666 can be obtained. The first *k* SGCs are constructed, respectively, and 1666 constructed signals can be obtained, where *k* = 1, 2, …, 1666. *PSE* of each constructed signal is computed, and the entropy distribution is displayed in Figure 12.

**Figure 13:**
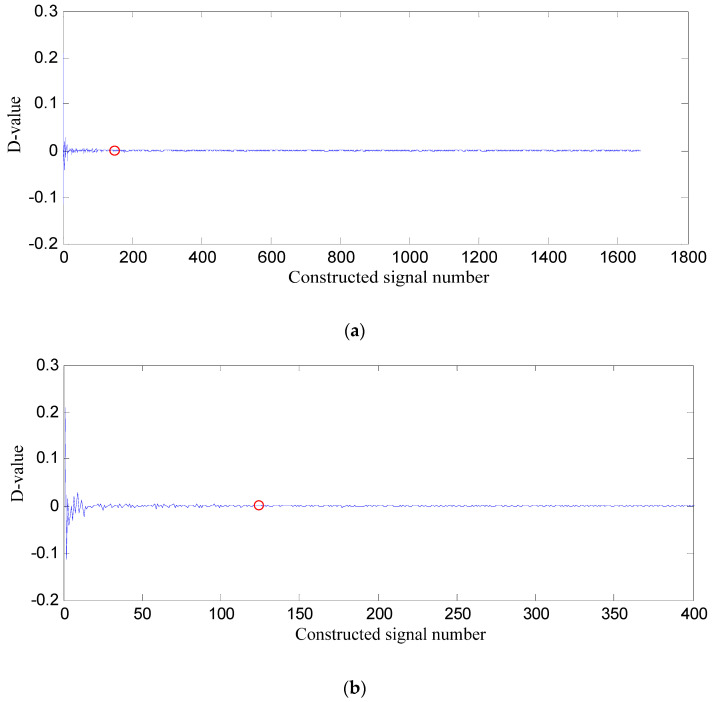
D-value distribution based on loose slipper fault signal. (**a**) All of constructed signal number; (**b**) The first 400 constructed signals.

In Figure 12, the analysis process is the same as that in Section 5.3. The *PSE* value of the former constructed signal subtracted from that of the latter one is difference value (D-value), and the D-value distribution is displayed in Figure 13. In Figure 13, D-values are much different in [0 166], and the values in [167 1665] are very close to 0, thus 166 is the turning point. The *PSE* value corresponding to 166 is not big and in the front range of *PSE* value, which means that the constructed signal of the first 166 SGCs may contain the richest fault feature information.

**Figure 14:**
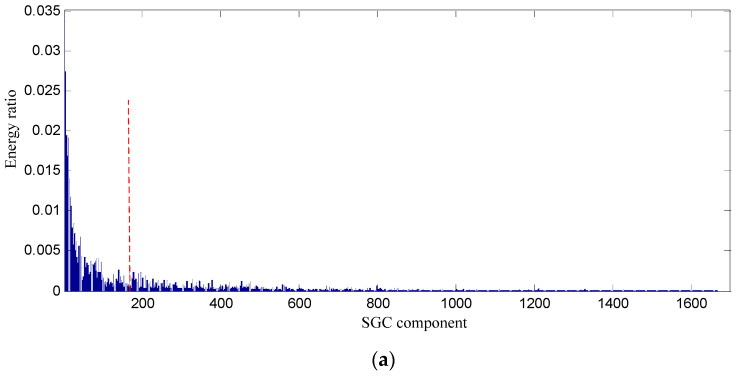

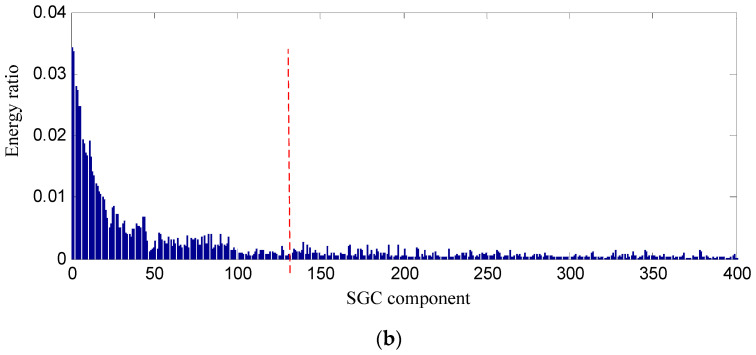
Energy distribution of each SGC component based on loose slipper fault signal. (**a**) All of components; (**b**) The first 400 components.

In order to verify the effectiveness of the proposed method of SGCs criterion, the energy ratio of SGC is adopted. The energy of each SGC component and the total energy of all components are computed, and energy ratio of each component energy to total energy can be obtained, and their distribution of 1666 components is displayed in Figure 14.

**Figure 15:**
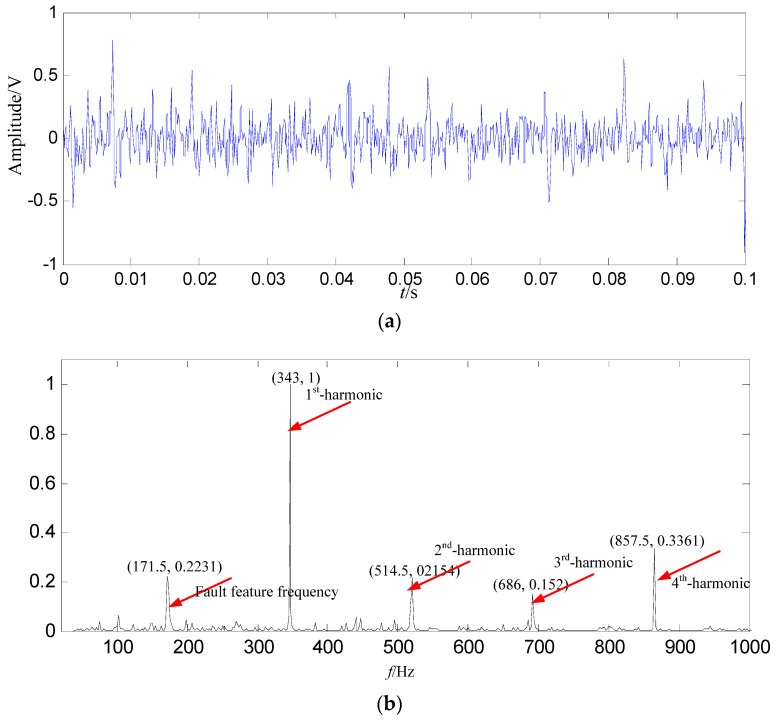
The constructed signal of the first 166 SGCs based on loose slipper fault signal. (**a**) Waveform in the time domain; (**b**) Power spectrum density in the frequency domain.

The conclusion can be drawn from the above that the constructed signal of the first 166 SGCs nearly involves all energy, thus the constructed signal contains the richest fault feature information. The waveform and power spectrum density of the constructed signal in the time and frequency domain are shown in Figure 15.

In Figure 15b, the fault feature information is at a fault feature frequency of 171.5 Hz and its harmonics are all extracted, and their fault feature information amount are very large, and there re nearly no interference components at other frequencies. Compared with the normal signal in Figure 3b, the fault feature information amount extracted from the constructed signal (the first 128 SGCs) is much larger. Compared with the loose slipper fault signal in Figure 11b, both of them are nearly the same, but the fault feature information is extracted only from the first 128 SGCs of all 1666 SGCs, and interferences and noises of 1538 SGCs are all effectively filtered by the proposed method.

#### 5.4.2. Application to Loose Slipper Fault Signal Based on EMD

**Figure 16:**
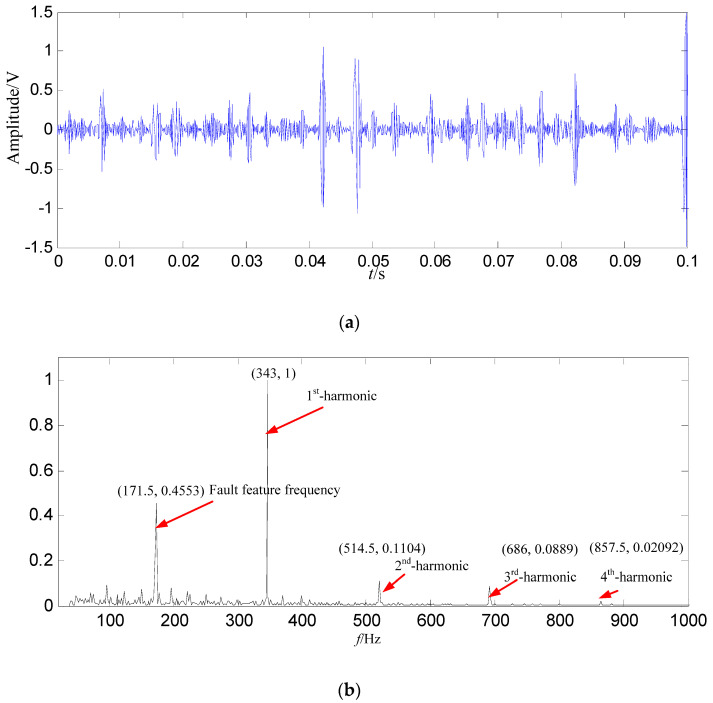
The EMD result based on loose slipper fault signal. (**a**) Waveform of *IMF*_1_ in the time domain; (**b**) Power spectrum density of *IMF*_1_ in the frequency domain.

In order to demonstrate the effectiveness and advantages of the proposed method, the signal is also decomposed by EMD. The energy of the first intrinsic mode function (IMF_1_) is the biggest among all IMFs, thus IMF_1_ is selected as the data source, and IMF_1_ is shown in Figure 16.

In Figure 16b, the fault feature information is mostly at a fault feature frequency of 171.5 Hz and its harmonics are all extracted, and the amount of fault feature information is large, and there are a lot of interference components at other frequencies. Compared with the constructed signal in Figure 15b, the fault feature information amount is less than those got by SGMD.

#### 5.4.3. Application to Loose Slipper Fault Signals Based on LMD

**Figure 17:**
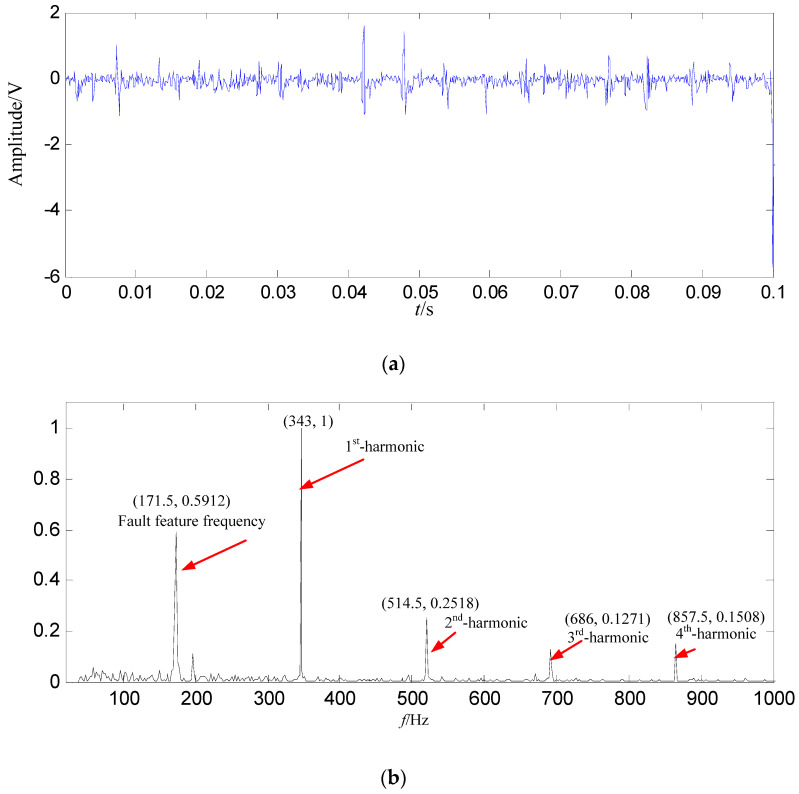
The LMD result based on loose slipper fault signal. (**a**) Waveform of *PF*_1_ in the time domain; (**b**) Power spectrum density of *PF*_1_ in the frequency domain.

LMD is also adopted to decompose the signal into several PFs, and the biggest energy corresponds to the first product function (PF_1_), and PF_1_ is used as the data source, and it is displayed in Figure 17.

In Figure 17b, the fault feature information is all extracted at a fault feature frequency of 171.5 Hz and its harmonics, but there are some interference components at other frequencies. Comparison analysis is implemented among the SGMD results in Figure 15b, EMD results in Figure 16b and LMD results in Figure 17b, where we can see the fault feature information amount is less than those obtained by SGMD, and is more than EMD result, furthermore there are more interference components in LMD result than those in the constructed signal.

It can be concluded from the above that: (1) SGMD can decompose the loose slipper fault signal effectively, and it is can be known that the first 128 SGCs contain the biggest amount of fault feature information based on the SGCs criterion. The amount of fault feature information obtained by SGMD is more than those obtained by EMD and LMD, thus the proposed method is superior to both EMD and LMD. (2) Compared with the normal signal in Figure 3a, loose slipper fault signal in Figure 11a, *IMF*_1_ in Figure 16a and *PF*_1_ in Figure 17a, although the fault signal, *IMF*_1_ and *PF*_1_ have periodic impact feature information in the time domain waveform, they are much interfered by the useless components.

### 5.5. Application to Center Spring Wear Fault Signal

#### 5.5.1. Application to Center Spring Wear Fault Signal Based on SGMD

**Figure 18:**
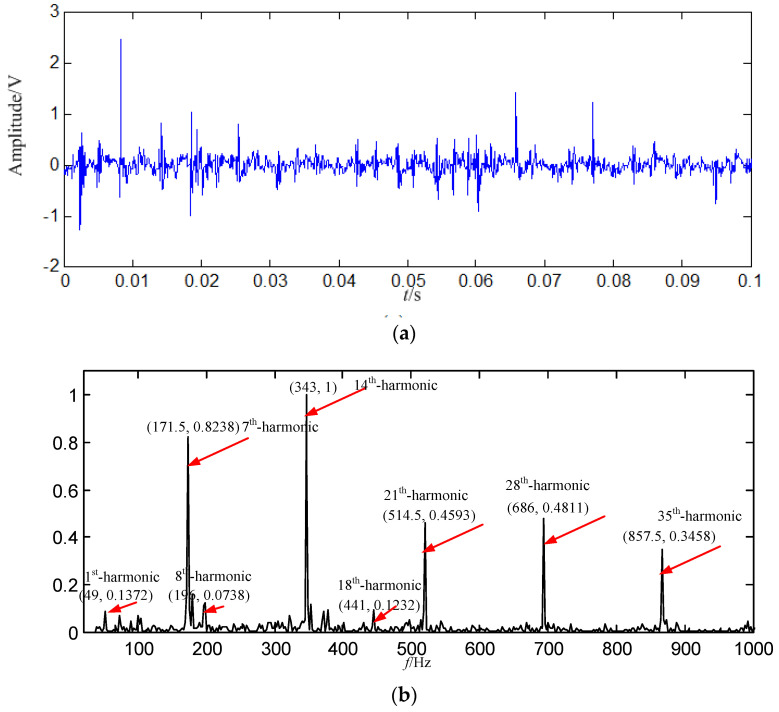
Center spring wear fault signal. (**a**) Waveform in the time domain; (**b**) Power spectrum density in the frequency domain.

A center spring wear fault signal with 5000 points (0.5 s) is decomposed in this study. In order display the signal clearly, the signal of 0.1 s is shown in Figure 18.

**Figure 19:**
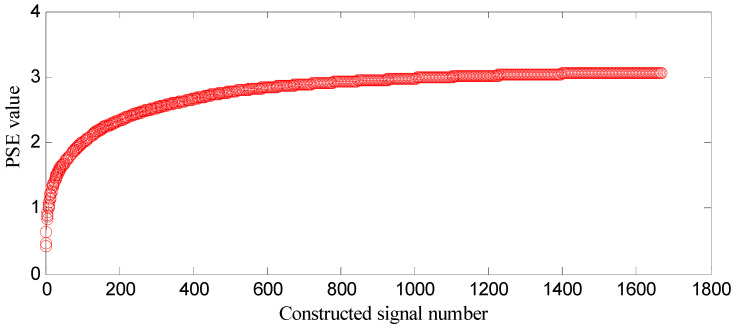
Entropy distribution based on center spring wear fault signal.

SGMD is applied to decompose the center spring wear fault signal, and SGCs of ⌊*d*⌋ = *n*/3 = 1666 can be obtained. The first *k* SGCs are constructed, respectively, and 1666 constructed signals can be obtainedt, where *k* = 1, 2, …, 1666. The PSE of each constructed signal is computed, and the entropy distribution is displayed in Figure 19.

**Figure 20:**
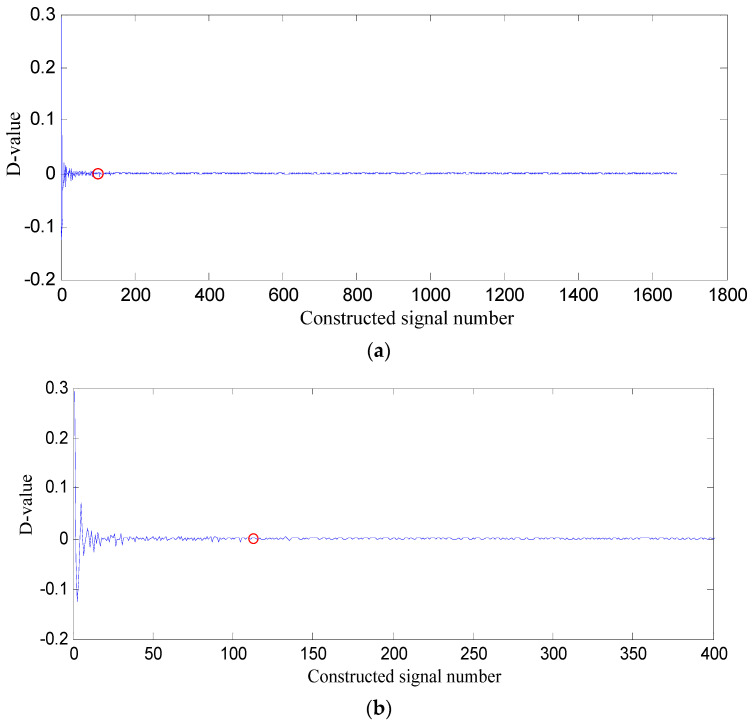
D-value distribution based on center spring wear fault signal. (**a**) All of constructed signal number; (**b**) The first 400 constructed signals.

In Figure 19, the analysis process is the same as that in Section 5.3. The PSE value of the former constructed signal subtracted from that of the latter one is the difference value (D-value), and the D-value distribution is displayed in Figure 20.

**Figure 21:**
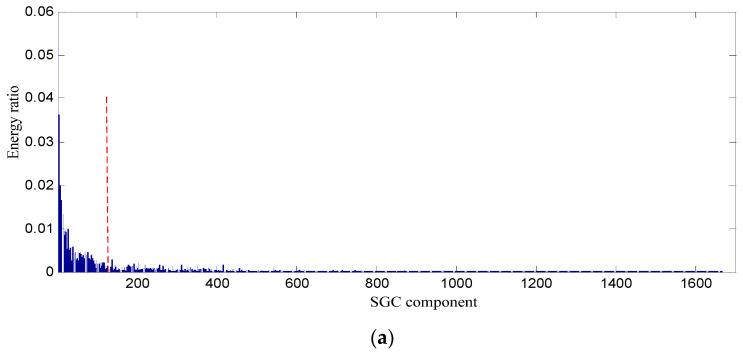

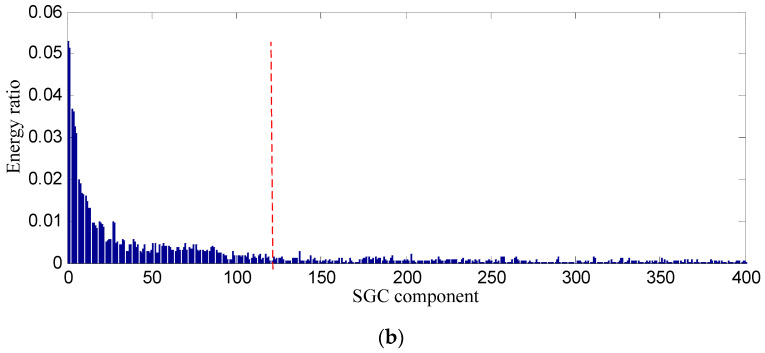
Energy distribution of each SGC component based on center spring wear fault signal. (**a**) All of components; (**b**) The first 400 components.

In Figure 20, D-values are much different in [0 128], and the values in [129 1665] are very close to 0, thus 128 is the turning point. The *PSE* value corresponding to 128 is not big and in the front range of *PSE* value, which means that the constructed signal of the first 128 SGCs may contain the richest fault feature information. In order to testify the effectiveness of the proposed method of SGCs criterion, the energy ratio of SGC is adopted. The energy of each SGC component and the total energy of all components are computed, and energy ratio of each component energy to total energy can be obtained, and their distribution of 1666 components is displayed in Figure 21.

**Figure 22:**
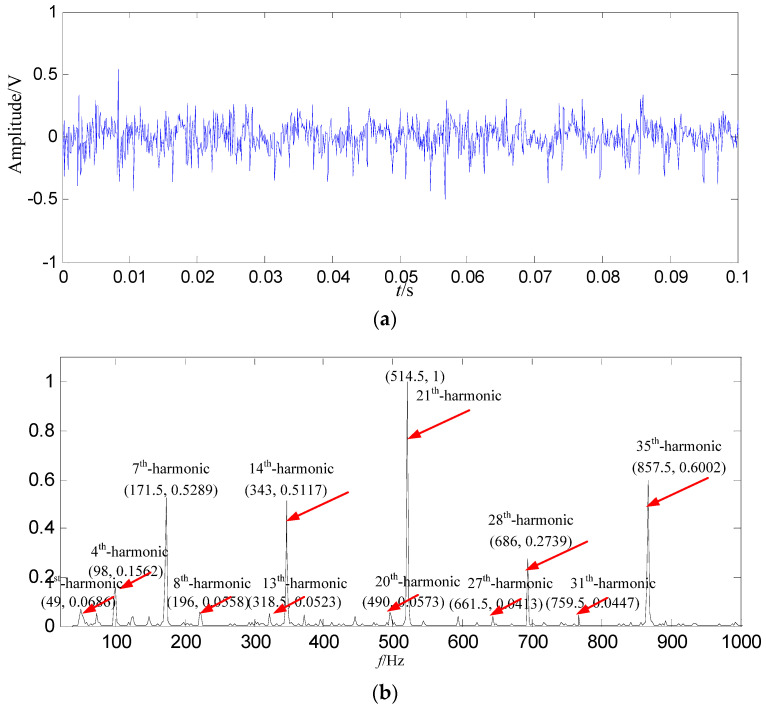
The constructed signal of the first 128 SGCs based on center spring wear fault signal. (**a**) Waveform in the time domain; (**b**) Power spectrum density in the frequency domain.

The conclusion can be drawn from the above that the constructed signal of the first 128 SGCs nearly involves all the energy, thus the constructed signal contains the richest fault feature information. The waveform and power spectrum density of the constructed signal in the time and frequency domain are shown in Figure 22.

In Figure 22b, most of the fault feature information is at a fault feature frequency of 24.5 Hz and its harmonics are nearly all extracted, and the amount of fault feature information is very large, and there are nearly no interference components at other frequencies. Compared with the normal signal in Figure 3b, the amount of fault feature information extracted from the constructed signal (the first 128 SGCs) is much larger. Compared with the center spring wear fault signal in Figure 18b, the fault amount of feature information extracted from the constructed signal (the first 128 SGCs) is a little larger.

#### 5.5.2. Application to Center Spring Wear Fault Signal Based on EMD

**Figure 23:**
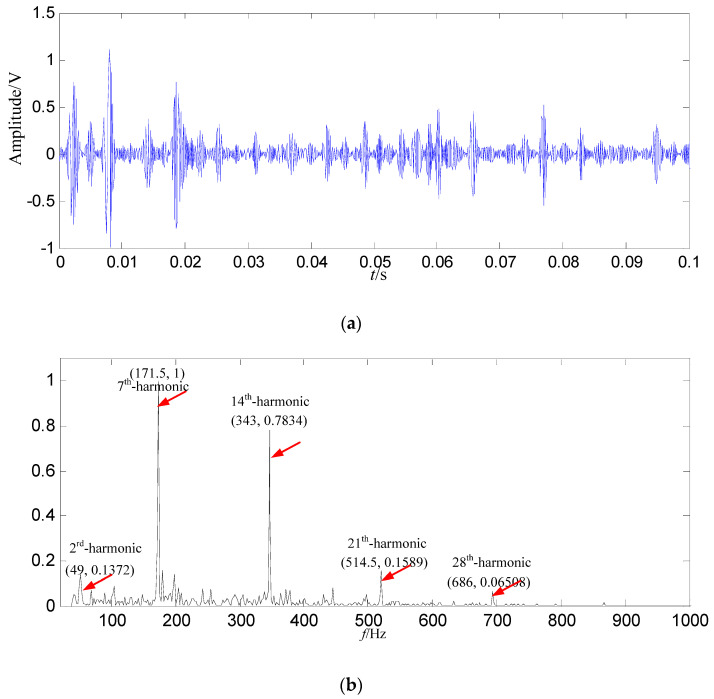
The EMD result based on center spring wear fault signal. (**a**) Waveform of *IMF*_1_ in the time domain; (**b**) Power spectrum density of *IMF*_1_ in the frequency domain.

In order to demonstrate the effectiveness and advantages of the proposed method, the signal is also decomposed by EMD. The energy of the first intrinsic mode function (IMF_1_) is the biggest among all IMFs, thus IMF_1_ is selected as the data source, and IMF_1_ is shown in Figure 23.

In Figure 23b, although some of the fault feature information at 171.5 Hz, 343 Hz, 514.5 Hz, 686 Hz and 857.5 Hz is extracted, most of the fault feature information ids at a fault feature frequency of 24.5 Hz and its harmonics are very small and even not extracted yet, and there are a lot of interference components at other frequencies. Compared with the constructed signal in Figure 22b, the fault feature information amount is less that obtained by SGMD.

#### 5.5.3. Application to Center Spring Wear Fault Signal Based on LMD

**Figure 24:**
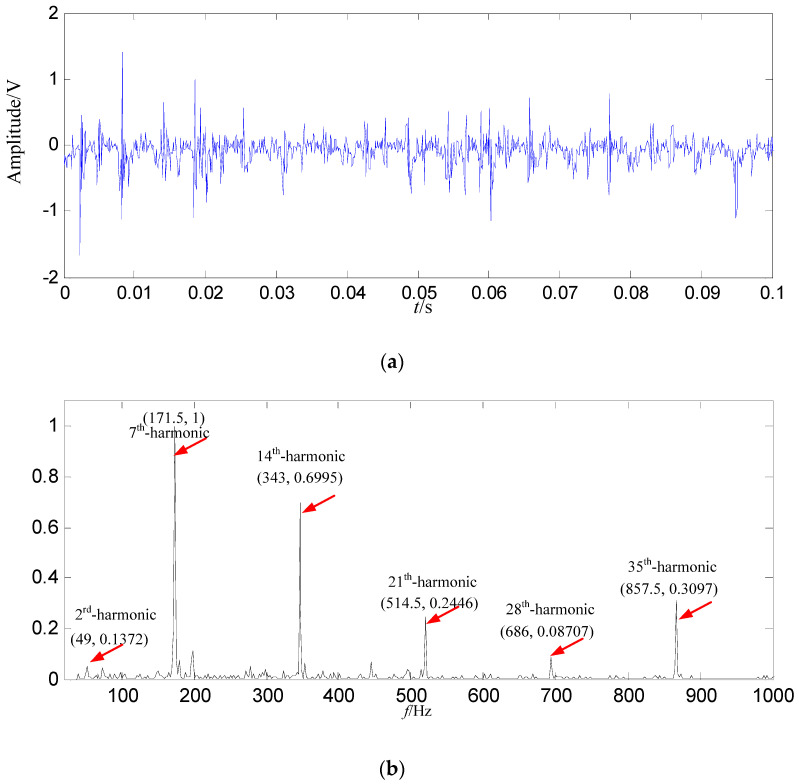
The LMD result based on center spring wear fault signal. (**a**) Waveform of *PF*_1_ in the time domain; (**b**) Power spectrum density of *PF*_1_ in the frequency domain.

The signal is decomposed into a series of PFs based on LMD. The biggest energy corresponds to *PF*_1_ in all of above PFs, thus the *PF*_1_ is used as the data source and is shown in Figure 24.

In Figure 24b, the fault feature information is extracted at a fault feature frequency of 24.5 Hz with only a few of its harmonics. The SGMD result in Figure 22b, EMD result in Figure 23b and LMD result in Figure 24b are all compared with each other, and it is can be seen that the amount of fault feature information obtained by LMD is more than those obtained by EMD, but it is much less than those got by SGMD.

Some conclusions can be drawn from the above: (1) SGMD can be used to decompose the center spring wear fault signal into some SGSs successfully, and the SGCs selection method criterion can be applied to pick up the first 128 SGCs which contain the richest fault feature information. (2) The fault feature information amount obtained by SGMD is more than those obtained by EMD and LMD, and the proposed method is more effective than the two methods. (3) Compared with the normal signal in Figure 3a, the center spring wear fault signal in Figure 18a, *IMF*_1_ in Figure 23a and *PF*_1_ in Figure 24a, the fault signal, *IMF*_1_ and *PF*_1_ have periodic impact feature information in the time domain waveform, but they are contaminated by interferences and noises.

After analysis of the above three kinds of hydraulic pump faults, it is can be concluded that the three kinds of fault signals can be decomposed by SGMD effectively, and about only top 10% of all SGCs (128 for swashplate wear fault, 166 for loose slipper fault, 128 for center spring wear fault) are extracted by the SGCs criterion based on PSE, and they have the largest amount of fault feature information and the proposed method is more effective than EMD and LMD.

## 6. Conclusions

Pointing at fault feature extraction of a hydraulic pump signal, a new method based on SGMD and PSE is proposed. The conclusions of the study are as follows:

(1) A multi-component fault signal can be decomposed into several SGCs by SGMD adaptively and effectively.
(2) SGCs criterion based on PSE is proposed, and it can extract several SGCs which contain the richest fault feature information to be reconstructed.
(3) The richest feature information is contained in about only top 10% of all SGCs.
(4) The proposed method performs better than EMD and LMD.

The advantages of the proposed method is that the SGCs can be selected by a SGCs criterion, and as much fault feature information as possible can be extracted, thus PSE can evaluate the complexity and uncertainty of measured information accurately. However, two remarks are noteworthy. First, since the number of SGCs is proportional to the signal length, while dealing with a long length signal, it may undergo low computational efficiency and serious overdecomposition. Second, because the PSE is very sensitive to useless interference and noise information, while encountering a heavy noise, it may be troublesome to effectively reflect the real complexity and uncertainty of the signal. In future works, the set of SGC numbers needs to be improved, and the set way should be more adaptive.

## References

[1] Lan Y., Hu J.W., Huang J.H., Niu L.K., Zeng X.H., Xiong X.Y., Wu B. (2018). Fault diagnosis on slipper abrasion of axial piston pump based on extreme learning machine. Measurement.

[2] Sun H., Yuan S., Luo Y. (2018). Cyclic Spectral Analysis of vibration signals for centrifugal pump fault characterization. IEEE Sens. J..

[3] Zhao Z., Jia M.X., Wang F.L., Wang S. (2009). Intermittent chaos and sliding window symbol sequence statistics-based early fault diagnosis for hydraulic pump on hydraulic tube tester. Mech. Syst. Signal Process..

[4] Du J., Wang S., Zhang H. (2013). Layered clustering multi-fault diagnosis for hydraulic piston pump. Mech. Syst. Signal Process..

[5] Lu C., Wang S., Makis V. (2017). Fault severity recognition of aviation piston pump based on feature extraction of EEMD paving and optimized support vector regression model. Aerosp. Sci. Technol..

[6] Teng W., Ding X., Zhang X., Liu Y., Ma Z. (2016). Multi-fault detection and failure analysis of wind turbine gearbox using complex wavelet transform. Renew. Energy.

[7] Dong W., Zhao Y., Yi C., Kwok-Leung T., Lin J.H. (2018). Sparsity guided empirical wavelet transform for fault diagnosis of rolling element bearings. Mech. Syst. Signal Process..

[8] Xin G., Hamzaoui N., Antoni J. (2018). Semi-automated diagnosis of bearing faults based on a hidden markov model of the vibration signals. Measurement.

[9] Lei Y., Zuo M.J., He Z.J., Zi Y.Y. (2010). A multidimensional hybrid intelligent method for gear fault diagnosis. Expert Syst. Appl..

[10] Feng Z., Chen X., Wang T. (2017). Time-varying demodulation analysis for rolling bearing fault diagnosis under variable speed conditions. J. Sound Vib..

[11] Chen H.X., Zuo M.J. Demodulation of gear vibration signals for fault detection. Proceedings of the 2009 8th International Conference on Reliability, Maintainability and Safety.

[12] Santos-Ruiz I., López-Estrada F.R., Puig V., Pérez-Pérez E.J., Mina-Antonio J.D., Valencia-Palomo G. (2018). Diagnosis of fluid leaks in pipelines using dynamic PCA. IFAC Pap. OnLine.

[13] Xiao Y., He Y. (2011). A novel approach for analog fault diagnosis based on neural networks and improved kernel PCA. Neurocomputing.

[14] Chen X., Xu X.Y., Liu A., Martin M. (2018). The use of Multivariate EMD and CCA for denoising muscle artifacts from few-channel EEG recordings. IEEE Trans. Instrum. Meas..

[15] Xiong Q., Xu Y.H., Peng Y.Q., Zhang W.H., Li Y.J., Tang L. (2017). Low-speed rolling bearing fault diagnosis based on EMD denoising and parameter estimate with alpha stable distribution. J. Mech. Sci. Technol..

[16] Yu D., Cheng J., Yang Y. (2005). Application of EMD method and Hilbert spectrum to the fault diagnosis of roller bearings. Mech. Syst. Signal Process..

[17] Feng Z., Dong Z., Zuo M.J. (2017). Adaptive mode decomposition methods and their applications in signal analysis for machinery fault diagnosis: A Review with Examples. IEEE Access.

[18] Shen Z.J., Chen X.F., Zhang X.L., He Z.J. (2012). A novel intelligent gear fault diagnosis model based on EMD and multi-class TSVM. Measurement.

[19] Dong H.B., Qi K.Y., Chen X.F., Zi Y.Y., He Z.J., Li B. (2009). Sifting process of EMD and its application in rolling element bearing fault diagnosis. J. Mech. Sci. Technol..

[20] Smith J.S. (2005). The local mean decomposition and its application to EEG perception data. J. R. Soc. Interface.

[21] Huang D., Ke L., Bo M., Zhao L. (2018). A new incipient fault diagnosis method combining improved RLS and LMD algorithm for rolling bearings with strong background noise. IEEE Access.

[22] Muruganatham B., Sanjith M.A., Krishnakumar B., Murty S.A.V.S. (2013). Roller element bearing fault diagnosis using singular spectrum analysis. Mech. Syst. Signal Process..

[23] Yi C., Yong L., Zhang D., Xiao H., Yu X. (2017). Quaternion singular spectrum analysis using convex optimization and its application to fault diagnosis of rolling bearing. Measurement.

[24] Safari N., Chung C.Y., Price G.C.D. (2018). A novel multi-step short-term wind power prediction framework based on chaotic time series analysis and singular spectrum analysis. IEEE Trans. Power Syst..

[25] Gu J., Lin P., Ling W.K., Yang C.Q. (2018). Grouping and selecting singular spectral analysis components for denoising based on empirical mode decomposition via integer quadratic programming. IET Signal Process..

[26] Floch Y.L., Pelayo Á. (2018). Symplectic Geometry and spectral properties of classical and quantum coupled angular momenta. J. Nonlinear Sci..

[27] Kang F. (1986). Difference schemes for Hamiltonian formalism and symplectic geometry. J. Comput. Math..

[28] Pan H.Y., Yang Y., Li X., Zheng J.D., Cheng J.S. (2019). Symplectic geometry mode decomposition and its application to rotating machinery compound fault diagnosis. Mech. Syst. Signal Process..

[29] Núñez J.A., Cincotta P.M., Wachlin F.C. (1996). Information entropy. Celest. Mech. Dyn. Astron..

[30] Ruizgómez S., Gómez C., Poza J., Gutiérrez-Tobal G.C., Tola-Arribas M.A., Cano M., Hornero R. (2018). Automated multiclass classification of spontaneous EEG activity in alzheimer’s disease and mild cognitive impairment. Entropy.

[31] Ji Y., Wang X., Liu Z., Yan Z., Jiao L., Wang D., Wang J. (2017). EEMD-based online milling chatter detection by fractal dimension and power spectral entropy. Int. J. Adv. Manuf. Technol..

[32] Llanos F., Alexander J.M., Stilp C.E., Kluender K.R. (2017). Power spectral entropy as an information-theoretic correlate of manner of articulation in American English. J. Acoust. Soc. Am..

[33] Jiang W., Zheng Z., Zhu Y., Li Y. (2015). Demodulation for hydraulic pump fault signals based on local mean decomposition and improved adaptive multiscale morphology analysis. Mech. Syst. Signal Process..

